# Removal of the Hazardous Congo Red Dye through Degradation under Visible Light Photocatalyzed by C,N Co-Doped TiO_2_ Prepared from Chicken Egg White

**DOI:** 10.1155/2022/2613841

**Published:** 2022-04-15

**Authors:** Nurul H. Aprilita, Della Amalia, Endang T. Wahyuni

**Affiliations:** Chemistry Department, Faculty of Mathematics and Natural Sciences, Universitas Gadjah Mada, Sekip Utara POB Bls 21, Yogyakarta, Indonesia

## Abstract

The C,N co-doped TiO_2_ photocatalyst was prepared by interacting the chicken egg white having various weights (1, 2, and 4 g) with 1 g of TiO_2_ in an autoclave through the hydrothermal process at 150°C. The C,N co-doped TiO_2_ photocatalysts were characterized using Fourier transform infrared (FTIR), X-ray diffraction (XRD), specular reflectance UV/visible (SRUV/Vis), and transmission electron microscope (TEM) instruments. The photocatalytic activity of the co-doped TiO_2_ was evaluated by monitoring the photo-decolorization of Congo red dye under visible light through a batch experiment. The characterization results assigned that the C and N atoms from the chicken egg white have been successfully co-doped into TiO_2_ through interstitial and substitutional combination, which could notably narrow their band gap energy entering into the visible region. In line with the gap narrowing, the co-doping C,N into TiO_2_ could remarkably improve the photocatalytic activity under visible light in the dye photo-decolorization. The enhancement of the photocatalyst activity of TiO_2_-C,N was controlled by the weight of the egg white introduced, and 2 g of the egg white resulted in the highest activity. Further, the best dye photo-decolorization, which was about 98%, of 10 mg/L Congo red dye in 100 mL of the solution under visible irradiation could be reached by applying TiO_2_-C,N prepared from 2 g of the egg white, within 45 min, at pH 7, and 50 mg of the photocatalyst mass.

## 1. Introduction

Titania (TiO_2_) is a photocatalyst with several excellent properties such as strong oxidative power, high chemical stability, low cost, and nontoxic [1–26], which has been widely used for the degradation of various toxic chemicals in light irradiation [2, 11, 18, 12–21, 25, 26]. TiO_2_ with the wide band gap energy (Eg), that is, 3.2 eV (for anatase type), however, has limited application since it can only be activated by UV light [1–3, 6, 8–10, 12] occupying a very small fraction (4–5%) in the solar spectrum [2, 6, 9, 10, 13, 14]. The other recognized weakness of TiO_2_ is the fast recombination of the electron-hole pair, which results in the low photocatalysis efficiency [6, 9, 10, 12, 13]. The application of TiO_2_ under cheap and abundant visible light and under solar light is beneficial that has to be afforded. In addition, the retardation of the electron-hole pair recombination in TiO_2_ is very essential, to get high photocatalysis efficiency.

An intensive effort has been directed to narrow the gap in the semiconductor structure of TiO_2_, that is by doping mono-nonmetallic elements including N [1, 3–10], S [2, 15], and C [12, 16], as well as double nonmetallic dopants, such as C-S [17], N-S [18, 19], N-P [14, 20], and C-N [13, 21–25]. All of the authors have found that the doping could noticeably enhance the visible light absorption and corresponding photocatalytic activity. In addition, some of them [4, 10, 14–17, 24] reported that the doping could not only broader the light absorption spectrum into the visible region to make it visible light active, but also promoted the separation of the hole-electron pair, which slowed down the recombination and so enhanced the efficiency of the photocatalysis process. The doping approach offers solutions for the two drawbacks of TiO_2_. Furthermore, compared with single-doped TiO_2_ photocatalysts, the doped TiO_2_ with two or more elements showed higher enhancement photocatalytic activity due to the beneficial synergy effect from the multi-dopants [14, 18, 21–24, 26].

Among the two dopant combinations, the double dopants of C-N are believed to be of high interest because it possesses both enhanced visible light absorption and separation efficiency of electron-hole pairs, which can contribute remarkably to improving photocatalytic activity [13, 21–25]. Many studies have focused on the preparation of C,N-doped TiO_2_, which frequently introduced two sources for the respective double dopants such as CCl_4_ and polyaniline [15], nitric acid and nonionic surfactant [22], and nitrate acid and acetylacetone [24]. Using a single source including diaminopyridine [13], polyaniline [21], and tetramethylammonium hydroxide [23] has gained a satisfactory result in constructing C,N co-doped TiO_2_. In comparison with the multisources, the single source has resulted in the more active co-doped TiO_2_ [18] due to the lesser residual of the dopant source left that impurified the TiO_2_ surface. In addition, the use of the single source of dopant is believed to be more practice and lower cost compared with the multisources [13, 18, 21, 23].

Many works have been addressed in the introducing single source for C,N dopants [13, 21, 23]; however, to the best of our knowledge, very limited study relates to protein in the chicken egg as the single source for multi-dopants on TiO_2_ [26]. A study [27] presented that the main components of the fresh chicken egg white are water (88.80%w), protein (10.60%w), carbohydrates (0.80%w), lipid (0.10%w), and very low sulfur (0.16%w). It is implied that large N and C atoms can be found in the egg white, allowing the egg white to be a potential single source for C and N multi-dopants.

Under the circumstance, in this study, the utilization of fresh chicken egg white as a single source of double dopants of C,N by hydrothermal method is addressed. We realize that a study on the co-doping TiO_2_ with C and N elements with a single source has been intensively conducted, but the use of the fresh chicken egg white is relatively new. A similar study has been reported [26], but this study dealt with the use of the expired chicken egg white as a single source for C,N,S triple dopants, and the co-doping was conducted by sol-gel method. The C,N,S co-doped TiO_2_ prepared was examined for rhodamine-B dye photodegradation.

In the current research, hydrothermal for co-doped TiO_2_ preparation is selected because this method involves high temperature, which was 124–200^o^C within 10–24 h [6], which meets with the temperature needed for decomposition of the compounds in the egg white. The egg white decomposition may result in the smaller compounds, which enable them to incorporate facilely in the TiO_2_ crystal lattice [2, 26].

**Figure 1:**
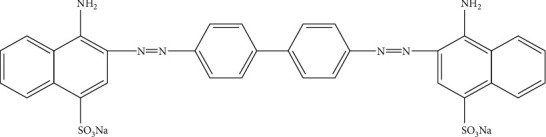
Chemical structure of Congo red dye.

The activity of the double co-doped photocatalyst in this work is investigated for Congo red (CR) dye removal from aqueous media. Congo red dyes, having a structure as seen in Figure 1, are selected as a pollutant probe due to their widely used for coloring cotton and other wearable materials [28–30]. The contamination of water by low levels of dye is noticeable, unpleasant, and hazardous for people's health and environment [30]. Consequently, the removal of the CR dye from wastewater is urgent to be conducted before reaching the environment. A favorite method for removing dyes is photodegradation, whether by undoped [30] and the doped TiO_2_ photocatalyst [28, 29], due to the effective destruction by forming harmless smaller molecules. To the best of our knowledge, no references regarding photodegradation of Congo red by TiO_2_ co-doped with C-N atoms from a single source of chicken egg white can be traced. Under the circumstance, in this study, the photodegradation of Congo red over the co-doped TiO_2_ under visible light is addressed. To obtain a condition giving maximum dye photodegradation, the photocatalyst dose, contact time, and solution pH are optimized.

## 2. Materials and Methods

### 2.1. Material

TiO_2_ P25, Congo red dye, HCl, and NaOH in the analytical grade were purchased from Merck Company and were used without any purification. Chicken eggs were collected from a public market in Yogyakarta, Indonesia.

### 2.2. Preparation of the Doped-TiO_2_ by Hydrothermal

In a typical process, 1 g of TiO_2_ suspended in water was mixed with chicken egg white with various weights (1, 2, and 4 g) accompanied by constant stirring at 500 rpm for 2 h. The mixture then was transferred into an autoclave to be heated in the oven at 180°C for 4 h. Afterward, the photocatalyst was collected, dried at 100°C for 2 h, and calcined at 500°C for 2 h. The samples obtained were coded as TiO_2_-C,N (1 : 1), TiO_2_-C,N (1 : 2), and TiO_2_-C,N (1 : 4) following the weight ratio of TiO_2_ powder to the egg white.

### 2.3. Characterization of the Co-Doped Photocatalysts

Characterizations of the undoped and co-doped TiO_2_ samples were conducted by several instruments, as explained below. The Fourier transform infrared (FTIR) spectra of the samples were recorded on a Shimadzu Prestige 21 FTIR spectrophotometer using the KBr pellet technique in the range of 4000–400 cm^−1^ to detect the functional group changes. PharmaSpec UV-1799 specular reflectance UV-visible (SRUV-visible) spectrophotometer was used to determine the Eg. A Shimadzu 6000X XRD powder diffractometer using Cu-K*α* radiation with 40 KV of the potential and 30 mA of the current was operated for crystallinity detection. The surface morphology of the co-doped TiO_2_ samples was observed by transmission electron microscope (TEM), which was taken on a JEOL JEM-2010 electron microscope operated at an accelerated voltage of 200 kV.

### 2.4. Photo-Decolorization of Dye Over TiO_2_-C,N

**Figure 2:**
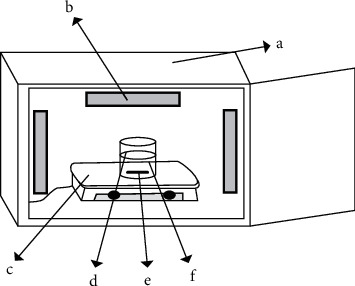
A set of apparatus used for photo-process, composed of (a) melamine box, (b) UV or visible lamps, (c) magnetic stirrer plate, (d) beaker glass, (e) magnetic stirrer bar, and (f) sample solution.

The dye photo-decolorization was conducted through a batch experiment in a set of closed apparatus (Figure 2) equipped with wolfram (visible light source) and/or deuterium lamp (UV light source) with TL-D intensity @20 W, 2000 lm/m^2^. A mixture of 100 mL of a solution containing 10 mg/L Congo red and 10 mg of the TiO_2_ powder was exposed under visible light for 45 min along with the constant rate of stirring. The solution from the photo-decolorization separated by filtration was analyzed by a UV-visible spectrophotometer at 510 nm of the wavelength to observe its absorbance. The absorbance observed then was interpolated into the respective standard curve, to get the dye concentration left in the solution. The dye photo-decolorization presented in % was calculated by following formula:

(1) $$\begin{array}{c} \%\text{photo}-\text{decolorization}=\frac{C_{0}-C_{1}}{C_{0}}\cdot 100\%, \end{array}$$

where Co is initial amount of Congo red (mg) and C_l_ is the amount of Congo red undegraded or left in the solution (mg).

The same procedure was duplicated for serial processes using TiO_2_-C,N (1 : 1), TiO_2_-C,N (1 : 2), and TiO_2_-C,N (1 : 4), as well as with various photocatalyst mass (10, 30, 50, 70, and 100 mg), variation of the irradiation time (5, 15, 30, 45, 60, 75, 90, and 120 min), and pH alteration (2, 4, 6, 8, and 10).

## 3. Results and Discussion

### 3.1. Characterization Data

#### 3.1.1. FTIR Data

**Figure 3:**
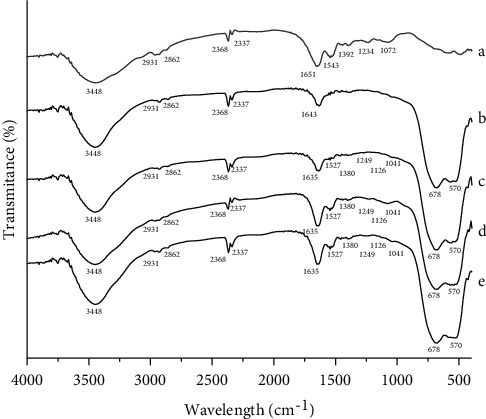
FTIR spectra of (a) chicken egg white, (b) TiO_2_, (c) TiO_2_-C,N (1 : 1), (d) TiO_2_-C,N (1 : 2), and (e) TiO_2_-C,N (1 : 4).

Figure 3 displays the FTIR spectra of the chicken egg white, TiO_2_, and all of the TiO_2_-C,N photocatalysts. The spectra of the chicken egg white (Figure 3(a)) present several absorption peaks located at 3448, 1651, 1543, 1234, and 1072 cm^−1^ that were attributed to O-H and/or N-H stretching vibration, the bending vibration of O-H bond, and/or C=O bonds from amide I in protein, the N-H bond of amide II in protein, the bond of C-N from amide III in protein, and the C-O bond vibration, respectively [31]. The several characteristic peaks suggest the presence of protein as the main component in the chicken egg white.

In the FTIR spectra of TiO_2_ (Figure 3(b)), the characteristic peaks are seen at 800 and 650 cm^−1^ that were attributed to Ti-O-Ti and O-Ti-O bond vibrations of the TiO_2_ lattice [1, 3, 15]. In addition, the broad absorbance peak located at 3400 cm^−1^ and the sharp peak at 1640 cm^−1^ are also observed, which were assigned to O-H stretching and bending vibrations, respectively, of water adsorbed on the surface of TiO_2_ [2, 8, 12, 13].

For the case of all co-doped TiO_2_-C,N samples, similar peaks to that of TiO_2_, with some additional new absorbance peaks, are observable. The new weak peak appears at 1527 cm^−1^ that could be ascribed to the N-H bond, as also found in the chicken egg white [31]. This peak suggests the presence of the residual of undecomposed protein from the egg. Another peak located at 1380 cm^−1^ indicated the presence of -NO_3_ absorbed [6] and/or -CO from -COO- of the amino acid group [31]. The peak corresponding to 1249 cm^−1^ was characteristic absorption of C-O bond [16] and/or of N-Ti bond [6]. The characteristic absorption spectrum of N-Ti bond is also seen at 1126 cm^−1^ [16]. The absorbance peak appearing at 1041 cm^−1^ was linked to the presence of Ti-C [16] and/or Ni-Ti [9]. The intensities of the presented peaks are seen to increase when the egg weights applied were enlarged. The appearance of the Ti-C and Ti-N bonds in the co-doped TiO_2_ suggests that C and N species have been incorporated into the TiO_2_ lattice, which may be through the substitutional of O atoms from TiO_2_ by C and N dopant atoms [3, 6]. The lasts are very weak peaks at 2901, 2862, 2368, and 2337 cm^−1^ that are observable in all samples. According to Lin [2], the weak peaks at 2368 cm^−1^ can be attributed to CO_2_ gas adsorbed on the TiO_2_ samples; meanwhile, the rest peaks were most possible from organic compounds polluting the KBr as pelleting material [2, 10].

#### 3.1.2. XRD Data

**Figure 4:**
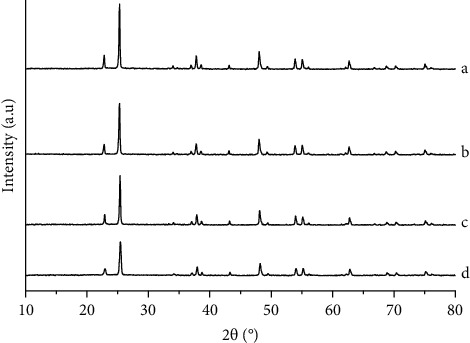
XRD patterns of (a) TiO_2_, (b) TiO_2_-C,N (1 : 1), (c) TiO_2_-C,N (1 : 2), and (d) TiO_2_-C,N (1 : 4).

The XRD patterns of C,N-doped TiO_2_ prepared with different quantities of chicken egg white, along with the un-co-doped TiO_2_, are demonstrated in Figure 4. All samples present diffraction peaks at 25.091, 37.651, 48.021, 53.891, 55.071, 62.381, 68.701, 70.041, and 75.001 of the 2*θ*, which are well fitted with those of the standard anatase phase of TiO_2_ recorded by JCPDS card number of 01-071-1167 [1, 4, 13].

The characteristic pattern of TiO_2_ crystal is noticeable in the XRD patterns of all TiO_2_-C,N samples, with no detectable dopant-related peaks. The figure also informs that the co-doping C,N into TiO_2_ leads to a decrease in the diffraction peak intensities, and the decrease is proportional to the weight of the egg white as the C,N dopant source. The decrease in the intensities implied a reduction in the crystallinity of TiO_2_ due to the partial structural distortion [10, 15]. It is also exhibited that the larger structural distortion of TiO_2_ occurred as the enhancement of the egg white weight since more C and N have been dopped. The distortion of the crystal could provide evidence that C and N from the white egg were successfully doped via substitutional mechanism [1, 3, 4, 6, 13].

#### 3.1.3. UV/Visible Reflectance Data

**Figure 5:**
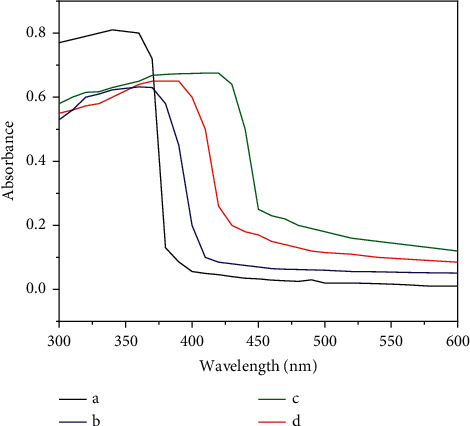
SRUV spectra of (a) TiO_2_, (b) TiO_2_-C,N (1 : 1), (c) TiO_2_-C,N (1 : 2), and (d) TiO_2_-C,N (1 : 4).

**Figure 6:**
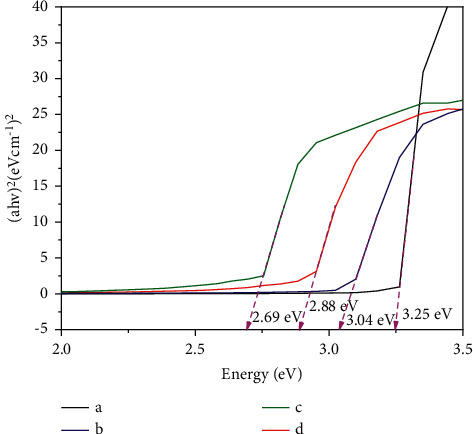
Tauc plot result of (a) TiO_2_, (b) TiO_2_-C,N (1 : 1), (c) TiO_2_-C,N (1 : 2), and (d) TiO_2_-C,N (1 : 4).

The UV/visible reflectance spectra of the co-doped photocatalysts are presented in Figure 5. Based on data in Figure 5, using the Tauc plot, the respective Eg values were obtained as illustrated in Figure 6. The absorption edge wavelengths and Eg values are displayed in Table 1. From Figure 5 and Table 1, it is apparent that the co-doping C and N atoms into TiO_2_ have remarkably shifted the light absorption into the visible region. The shift was created by narrowing the gap in the TiO_2_ semiconductor structure [4, 6, 13, 15, 18, 19], due to the double atoms incorporated in the lattice of TiO_2_ crystal [6]. Furthermore, the decrease in Eg, as seen in Figure 6 and Table 1, was found to be more effective when the amount of the dopant source was enlarged since more amount of C and N dopants could occupy the gap. However, with the largest mass of the chicken egg white, a decrease in the band energy was less effective. This opposite trend can be caused by the agglomeration of the organic material from the chicken egg white, which constrains to insert into the lattice crystal of TiO_2_. The same trend was also reported previously [4, 13, 15, 18, 19]. The significant decrease in the Eg values provides evidence of the interstitial C and N co-doped mechanisms [3, 6, 10].

#### 3.1.4. The TEM Data

**Figure 7:**
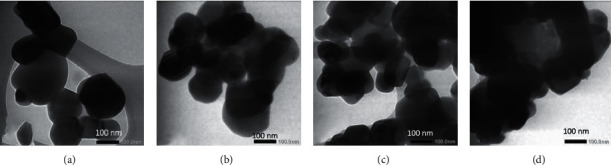
TEM images of (a) TiO_2_, (b) TiO_2_-C,N (1 : 1), (c) TiO_2_-C,N (1 : 2), and (d) TiO_2_-C,N (1 : 4).

The TEM images of undoped and the co-doped TiO_2_ samples are displayed in Figure 7. It is seen that TiO_2_ particles have a spherical shape of various sizes. In the TiO_2_-C,N samples, the spherical shapes are seen as darker due to the C,N co-doped into TiO_2_ lattice. A similar image has also been obtained [24]. The large agglomerates coating the TiO_2_-C,N surface are observed when a very large weight (4 g) of the egg white was introduced. With the very large amount of the egg white, the protein in the egg white may be incompletely decomposed, resulting in the large organic compound. The large compounds forming agglomerate [2] blocked the TiO_2_ surface. These TEM images imply the occurrence of the co-doping C and N from the chicken egg white to the TiO_2_ crystal [25].

### 3.2. Photocatalytic Activity of the Doped TiO_2_-C,N

#### 3.2.1. The Effect of Co-Doping on the TiO_2_-C,N Photocatalytic Activity

**Figure 8:**
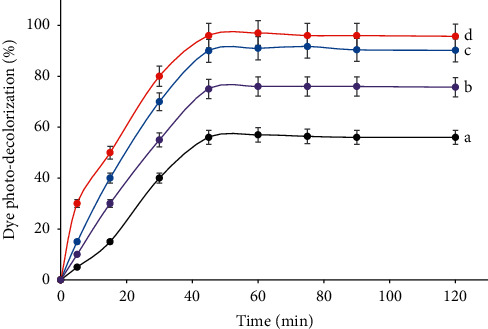
Dye photo-decolorization results, in the presence of (a) TiO_2_ + visible, (b) TiO_2_ + UV, (c) TiO_2_-C,N (1 : 2) + visible, and (d) TiO_2_-C,N (1 : 2) + UV (Congo red dye concentration = 10 mg L^−1^, solution volume = 100 mL, photocatalyst mass = 50 mg, reaction time = 45 min, and pH = 7).

The activity of the co-doped photocatalyst represented by TiO_2_-C,N (1 : 2) was evaluated through Congo red photo-decolorization. The effect of the co-doping on the activity of TiO_2_-C,N under UV and visible light exposure is presented in Figure 8.

Figure 8(c) reveals, as expected, that the co-doping has improved the photo-activity of TiO_2_-C,N in the dye photo-decolorization under visible light irradiation, in comparison with the un-co-doped one (Figure 8(a)). Under visible light irradiation, the dye photo-decolorization over undoped TiO_2_ was around 57% and the dye photo-decolorization with TiO_2_-C,N increased up to 91%. The photocatalyst of TiO_2_-C,N (1 : 2) with Eg as high as 2.67 eV that is equal to the energy of visible light allowed it to strongly absorb the visible light, which could generate an adequate number of OH radicals represented by equation (2) until equation (5) [2]. The radicals played a very important role in the dye decolorization since the radicals can act as strong oxidation agent [11], which was able to destroy the Congo red effectively into smaller molecules such as CO_2_, H_2_O, and NO_3_^−^ [28, 30]. In contrast, the bare TiO_2_ with Eg of 3.25 eV (Figure 8(a)), which is higher than the energy of the visible light, was constrained to release electrons from the valence band under visible stimulation [2, 3], and so only a few numbers of OH radicals could be provided. Accordingly, the higher effectiveness of the dye photo-decolorization of co-doped TiO_2_ over un-co-doped TiO_2_ resulted. A similar trend has been reported by many studies [4, 13, 15, 18].

(2) $$\begin{array}{c} {\text{TiO}}_{2}+\text{light}⟶{\text{TiO}}_{2}\left(e^{-}+h^{+}\right), \end{array}$$

(3) $$\begin{array}{c} h^{+}+{\text{H}}_{2}\text{O}⟶OH+H^{+}, \end{array}$$

(4) $$\begin{array}{c} \text{TiOH}+h^{+}⟶Ti\cdot OH, \end{array}$$

(5) $$\begin{array}{c} h^{+}+O_{2}⟶O_{2}. \end{array}$$

The dye photo-decolorization process under UV light over TiO_2_-C,N (1 : 2) (Figure 8(d)) is much more effective than over the bare TiO_2_ (Figure 8(b)). It is clear that the co-doping also enhanced the TiO_2_ activity under UV light, as also found by others [4, 10, 13, 15–17]. The enhancement should be promoted by inhibiting the electron-hole recombination since the dopants can act as a separation center [4, 10, 13, 15–17].

#### 3.2.2. The Influence of the Dopant Loaded in TiO_2_

**Figure 9:**
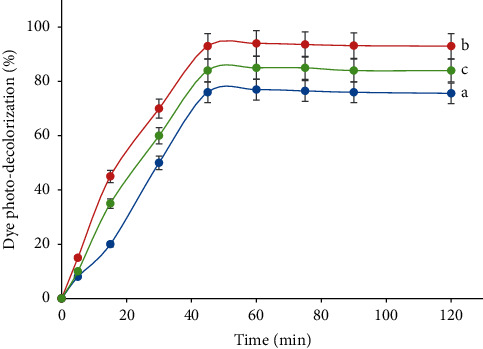
Dye photo-decolorization efficiency under visible process over TiO_2_-C,N prepared from the egg white with (a) 1 g, (b) 2 g, and (c) 4 g into 1 g TiO_2_ (Congo red dye concentration = 10 mg L^−1^, solution volume = 100 mL, photocatalyst mass = 50 mg, reaction time = 45 min, and pH = 7).

The influence of the egg weight introduced into TiO_2_ towards the activity of the co-doped TiO_2_ in the dye decolorization is displayed in Figure 9. Concerning the C,N-doped samples, an increase in the activity is observed for all samples compared with the corresponding pure TiO_2_ materials. Further, it is seen that the increase in the egg weight resulted in higher dye decolorization, but larger egg weight than 2 g caused opposite photo-decolorization result. Many studies have also found a similar trend [4]. The mass of the egg white should be proportional to the amount of C, N co-doped into TiO_2_. The photocatalyst with the increasing amount of the dopants, having lower Eg, was able to absorb visible light more effectively. This condition provided more number of radicals, which further resulted in the higher dye photo-decolorization.

The heaviest weight of the egg white, implying the largest dopant content, should result in the highest effective photo-decolorization, but the contrary result was observed. In this typical co-doped TiO_2_, the agglomerate of the organic residual covered the TiO_2_ surface, as demonstrated by TEM images, which limited the contact of TiO_2_-C,N (1 : 2) with the light, diminishing OH radical formation. In addition, the covered surface of TiO_2_ also inhibited the OH radical on the TiO_2_ surface to contact with the dye. These situations constrained the dye photo-decolorization.

From Figures 8 and 9, it is notable that the photo-decolorization is enhanced sharply as the length of the irradiation time up to 30 mins, then the slight increase is notified within 35 to 45 mins, but with a longer time than 45 mins, the photo-decolorization is not influenced by time. Similar data have also been reported by others [2]. The extension time could enhance the light penetration to reach the TiO_2_ surface, which resulted in a larger number of OH radicals, and so that a greater collision frequency occurred between the OH radicals and the dye [2]. These conditions were conducive to dye photo-decolorization. After reaching the maximum formation of the OH radicals and so the photo-decolorization, TiO_2_-C,N may be saturated that suffered from the formation of the OH radicals, so that the photo-decolorization insignificantly changed or almost constant [2, 10].

#### 3.2.3. The Influence of the Photocatalyst Mass and Solution pH

**Figure 10:**
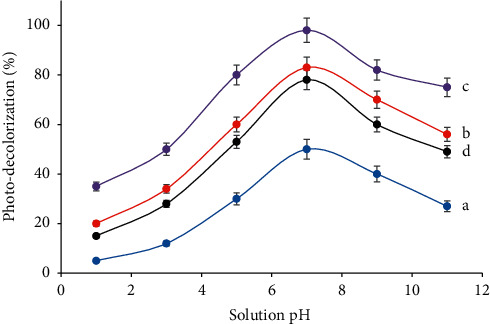
Influence of pH on the photo-decolorization, with the photocatalyst mass of (a) 10 mg, (b) 30 mg, (c) 50 mg, and (d) 100 mg in 100 mL of the dye solution (Congo red dye concentration = 10 mg L^−1^, solution volume = 100 mL, egg white mass = 2 g, and reaction time = 45 min).

The photo-decolorization resulting from the process with various photocatalyst mass and pH alteration is displayed in Figure 10. In the figure, more effective photo-decolorization could be reached with the enlargement of the photocatalyst mass, but with the higher mass than the optimum level, the photo-decolorization was found to be detrimental. With increasing photocatalyst mass, more OH radicals were provided, hence improving the dye photo-decolorization. In contrast, a very large mass caused more turbid solution, which screened the light penetration, and so the photo-decolorization was retarded [14, 10].

It is also observable that the photo-decolorization significantly improved when the pH was elevated and reached the maximum level at pH 7. The opposite trend appears at pH higher than 7. At low pH, the surface of TiO_2_ was protonated by H^+^ to form positively charged surface [14, 10] and Congo red also existed as cationic form [20, 21]. The same charges refused the interaction between TiO_2_ surface and Congo red dye molecules, which led to the photo-decolorization being very less effective. It is important to note that most OH radicals are associated with TiO_2_ surface and for proceeding with the photo-decolorization, and Congo red has to be adsorbed on the surface of the photocatalyst to interact with the OH radicals [12, 19]. Increasing pH can gradually reduce the protonation of both photocatalyst [12] and Congo red molecules [20] to form neutral molecules. The increase in the number of neutral molecules is beneficial to mutually interact, which promoted more effective photo-decolorization. In the solution with higher pH (basic condition), both the surface of TiO_2_ and the Congo red tended to be negatively charged [12, 20]. Consequently, the interaction between Congo red and the TiO_2_ photocatalyst was constrained, causing the photo-decolorization declined as shown in Figure 10.

## 4. Conclusions

It can be concluded that chicken egg white as a single source can be used for co-doped C,N into TiO_2_ that has successfully decreased the Eg and shifted the absorption into the visible region. The decrease in Eg was influenced by the amount of the chicken egg, and the highest decrease in Eg (into 2.69 eV from 3.25 eV) was demonstrated by the photocatalyst of TiO_2_-C,N prepared from 2 g of the chicken egg white for 1 g of TiO_2_. Further, the doped TiO_2_-C,N exhibited stronger activity in the photo-decolorization of Congo red dye under visible irradiation than the undoped photocatalyst did. The highest dye photo-decolorization in 100 mL of the solution with 10 mg/L of the concentration could be reached using TiO_2_-C,N (1 : 2) photocatalyst with 50 mg of the mass, for 45 min and at pH 7, which was about 98%.

## Figures and Tables

**Table 1:** Effect of doping on the band gap energy of TiO_2._

Sample	TiO_2_	TiO_2_-C,N (1 : 1)	TiO_2_-C,N (1 : 2)	TiO_2_-C,N (1 : 4)
The absorption wavelength (nm)	387	415	450	430
The band gap energy (eV)	3.25	3.04	2.69	2.88

## Data Availability

The characterization data including X-ray diffraction, Fourier transform infrared, specular reflectance UV/visible, and transmission electron microscope used to support the findings of this study are included within the article. The efficiency of the dye photo-decolorization data used to support the findings of this study are included within the article.
